# Simultaneous multi-band valley-protected topological edge states of shear vertical wave in two-dimensional phononic crystals with veins

**DOI:** 10.1038/s41598-017-10857-2

**Published:** 2017-09-04

**Authors:** Shao-yong Huo, Jiu-jiu Chen, Hong-bo Huang, Guo-liang Huang

**Affiliations:** 1 State Key Laboratory of Advanced Design and Manufacturing for Vehicle Body, College of Mechanical and Vehicle Engineering, Hunan University, Changsha, 410082 People’s Republic of China; 2 Department of Mechanical and Aerospace Engineering, University Misssouri, Columbia, MO 65211 USA

## Abstract

The introduction of the concept of valley pseudospin to phononic crystals has made a remarkable topologically protected interface transport of sound, which opens a novel research area referred to as valley Hall topological insulators. Here, we demonstrate the simultaneous multi-band edge states of shear vertical waves in two-dimensional phononic crystals with veins. The multi-band edge states are topologically valley-protected and are obtained by simultaneously gapping multiple Dirac points at K (or K′) under the inversion symmetry breaking. As the relative radius of the two adjacent steel columns varies, the band diagram undergoes a topological transition which can be characterized by topological charge distributions and opposite valley Chern numbers. Subsequently, the vortex chirality of the bulk valley modes is unveiled. With numerical simulations, simultaneous multi-band valley dependent edge states and the associated valley-protected backscattering suppression around the curved waveguide are further demonstrated. Our work could become a promising platform for applications of multi-functional topological acoustic devices.

## Introduction

The discoveries of quantum Hall effect^1^, quantum spin Hall effects^2, 3^, and topological insulators^4, 5^ have opened a new door for the understanding of condensed matter physics. In recent years, inspired by these concepts, the exploration of the topological properties of classical waves has been a hot research topic in photonic crystals and optomechanics areas^6–9^. The topological properties describing the quantized behavior of wavefunctions over associated dispersion bands have been found to have a profound influence on transportation properties in electronic systems such as Haldane lattices and grapheme structures^10^. Then, the concept of topology was analogously applied to the classical waves to provide a new degree of freedom in controlling and manipulating photons, plasma-waves, and electromagnetic waves^11–13^. The exploration of topology in photonic crystals and metamaterials with non-zero-gauge fields has motivated a number of intriguing optical phenomena such as one-way transport and Weyl physics^14, 15^.

As one of the classical waves, acoustic wave can be scattered or diffracted when suffering inhomogeneity in the propagation path, which offers an effective routes to steer the acoustic waves by some neoteric artificial structures, such as acoustic metamaterials^16, 17^, metasurfaces^18^ and parity-time symmetry medium^19, 20^. Recently, analogous to the topology mechanism in electron and photonic system to control wave propagation, the concepts of topology based on the quantum Hall effect have also inspired a novel field of “topological acoustics”^21–37^. The topological properties of one-way elastic edge state^22^, acoustic topological insulator^23^, and topological valley transport^24^ that are immune to backscattering in the presence of imperfections and impurities including localized defects and sharp corners are being studied by some scientists. Achieving these topologically protected acoustic propagations is mainly depending on two categories of mechanisms. The first is to break time-reversal symmetry by the active components or the application of external fields. By introducing a circulating fluid flow^25–27^ or rotating gyroscopes^22^ to break the time-reversal symmetry, the acoustic topologically nontrivial edge states with strong robustness were demonstrated in nonreciprocal acoustic systems. The other mechanism exhibits the topological edge modes in analogy with the quantum spin Hall effects. Based on the acoustic quantum spin Hall effect, the robust pseudospin-dependent one-way edge sound transport^23, 28–30^ has been successfully demonstrated in time-reversal invariant systems. Meanwhile, the concept of valley pseudospin, labeling quantum states of energy extrema in momentum space, is being explored by some researchers. Lu *et al*. have reported the experimental observation of topological valley transport of sound by breaking rotating symmetry relying on the quantum valley Hall effects^24, 31^. However, most of the aforementioned studies have realized the topological protected edge states in fluid medium where the acoustic waves are purely longitudinal. The research of topological physics in solid domains is extremely challenging since it exists as a transverse polarization mode and a mixed (longitudinal-transverse) polarization mode in solid phononic crystals. Moreover, the multi-band topological protected edge states have not been investigated, which could be more promising and convenient for practical applications of multi-functional topological acoustic devices. Therefore, it is essential and significant to explore the simultaneous multi-band valley-protected topological edge states of the shear vertical (SV) waves, which will greatly enrich the knowledge on the topology and its applications.

Here, we present a design to realize the simultaneous multi-band valley-protected topological edge states for SV waves in two-dimensional phononic crystals with veins. Through a simple inversion-symmetry-breaking, multiple Dirac points at K (or K′) are simultaneously split. We demonstrate the existence of topological transition as the radii of the two adjacent steel columns varies, which is characterized by topological charge distributions and opposite valley Chern numbers. In addition, the chiral characteristic of the bulk valley modes is clearly presented. Through the numerical simulations of band structures, we confirm the appearance of simultaneous multi-band valley-protected topological edge states. Furthermore, valley-protected backscattering suppressions of SV waves propagating along the straight waveguide and the curved waveguide are demonstrated in multiple frequency ranges.

## Results

### Phononic crystal design and band structures

**Figure 1:**
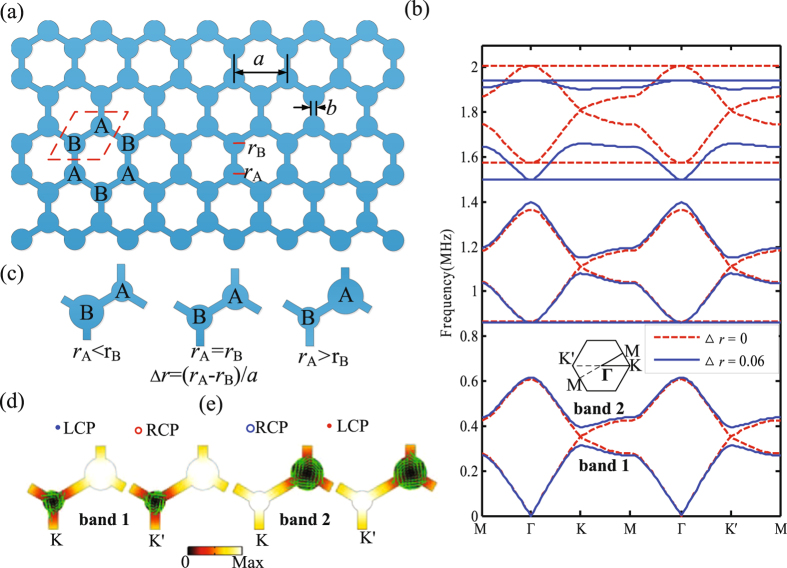
Band structure for the two-dimensional phononic crystal with veins and the vortex profiles of the bulk valley modes. (**a**) Schematic of the two-dimensional phononic crystals with veins. The unit cell consists of two steel rods connected by slender veins in honeycomb lattice. (**b**) The band structures for Δ*r* = 0 (red dashed line) and Δ*r* = 0.06 (blue solid line). Multiple Dirac cones appear at two inequivalent but time-reversal valleys (K and K′) and they are lifted when the inversion symmetry is broken. (**c**) The evolution of the basic unit cell as the Δ*r* varies. (**d**,**e**) The stress field distributions and the vortex profiles of the bulk valley modes at K and K′ valley for band 1 and band 2, respectively.

The physical design, based on the regular honeycomb lattice with circular steel rods connected by slender veins, is shown in Fig. 1(a). The red dashed outline shows the basic unit cell. The structural geometry can be described by the lattice constant *a* (=4 mm), the radius of the steel rods *r* (*r*_A_ = *r*_B_ = 0.55 mm) and the vein width *b* (=0.4 mm). The material parameters of steel are chosen as follows: the elastic modulus *E*_*steel*_ = 210.6 GPa, the shear modulus *μ*_*steel*_ = 81 GPa, and the density *ρ*_*steel*_ = 7780 kg/m^3^. We calculate the band structures of SV waves in this phononic crystal by using the finite element method (FEM) with COMSOL Multiphysics. From Fig. 1(b), it can be seen that three pairs of two-fold Dirac point degeneracies are formed at K (or K′) point for *r*_A_ = *r*_B_, indicated by the red dashed line, which is due to the fact that the conical dispersions at K (K′) valleys are protected under the time-reversal symmetry and spatial inversion symmetry. To break the inversion symmetry, a perturbation is introduced by varying the relative radius of steel rods *r* in the unit cell. For convenience, we define the difference between rod A and rod B as the parameter Δ*r* (Δ*r* = (*r*_A_ − *r*_B_)/*a*) to characterize the breaking of inversion symmetry as shown in Fig. 1(c). Meanwhile, the average radius *r*_*a*_ = (*r*_B +_ *r*_A_)/2 is kept constant (*r*_*a*_ = 0.55 mm) when the Δ*r* varies. As shown in Fig. 1(b), it is observed that three pairs of two-fold Dirac point degeneracies at the K (or K′) valley are simultaneously split out when the parameter Δ*r* is 0.06. According to the view of point group, when the Δ*r* is altered, the symmetry of the K (and K′) point is reduced from *C*_3*v*_ to *C*_3_. Thus, the degenerate irreducible representation *E* will transform into two non-degenerate irreducible representations *E*_1_ and *E*_2_, which is also associated with band inversion process^38^.

To reveal the band inversion process and topological property transition, we take the first pair of Dirac point degeneracies for example to illustrate it. Firstly, the stress field distributions and the vortex chirality of the bulk valley modes of SV waves are clearly presented for the parameter Δ*r* = 0.06. As shown in Fig. 1(d) and (e), the valley modes of band 1 and band 2 exhibit typical vortex profiles centered at the two inequivalent circles. At the K point, the energy flux (arrow pointing) of band 1 (band 2) shows the anticlockwise (clockwise) vortex profiles concentrated at the smaller (larger) radius. Similarly, at the K′ point, the energy flux (arrow pointing) of band 1 (band 2) displays the opposite chirality. This attractive chirality characteristic of the bulk valley modes of SV waves originates from the fact that every valley (K and K′) modes of the Dirac band (band 1 and band 2) carry intrinsic orbital angular momentum^31, 39^. Here, we define the anticlockwise vortex profiles at the K point for band 1 as a left circular polarized (LCP) orbital angular momentum and the clockwise vortex profiles at the K′ point for band 1 as a right circular polarized (RCP) orbital angular momentum. The other two valley modes can be similarly identified. From Fig. 1(d) and (e), it is worth noting that the energy fields are weak at the place where the stress fields are strong, which is mainly due to the fact that the energy flux is simultaneously modulated by the stress fields and phase gradient^31^.

**Figure 2:**
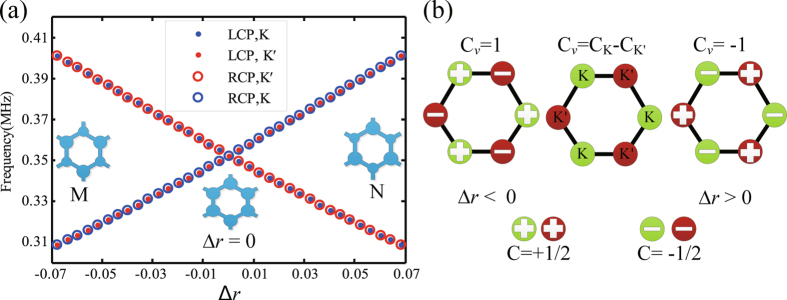
The topological transitions, topological charge distributions and valley Chern numbers. (**a**) The evolution of valley modes (blue dots and circles for K valley and red dots and circles for K′ valley) as a function of Δ*r*. The left inset (defined as M type) corresponds to Δ*r* = −0.06 and the left inset (defined as N type) corresponds to Δ*r* = 0.06. The middle inset corresponds to Δ*r* = 0. (**b**) The topological charge distributions and nonzero valley Chern numbers for phononic crystals with Δ*r* < 0 and Δ*r* > 0.

Then, the topological transition in the evolution of the inversion asymmetry parameter Δ*r* is further analyzed. Figure 2(a) shows the evolution of valley modes as a function of Δ*r*. With the increase of Δ*r*, the radius of rod A becomes larger but that of rod B which becomes smaller. As a result, the modes whose stress fields are concentrated on the A (B) rods will have lower (higher) frequency. Furthermore, the frequency spectra for the K valley mode of the LCP and the K′ valley mode of RCP decrease monotonously, while the frequency spectra for the K′ valley mode of the LCP and K valley mode of RCP rise up gradually as the increase of Δ*r*, in which band 1 and band 2 undergo the band gap closing and reopening. When the parameter Δ*r* is equal to zero, crossing of the two bands appears. Meanwhile, the exchange between the modes at the same valley occurs, which leads to the topological transition. In order to have deeper understanding of the topological transition, we introduce the viewpoint of a nonzero valley Chern number. Through the **k**•**p** perturbation method, we can obtain the effective Hamiltonian^24^

1 $${H}_{K}(\delta {\boldsymbol{k}})={\nu }_{D}\delta {k}_{x}{\sigma }_{x}+{\nu }_{D}\delta {k}_{y}{\sigma }_{y}+m{\nu }_{D}^{2}{\sigma }_{z},$$

where ν_*D*_ is the Dirac velocity of the conic dispersion. *δ* ***k*** is the momentums deviation from the valley center K. *σ*_*i*_ are the Pauli matrices of the vortex pseudospins. More importantly, the mass term of $m={\rm{\Delta }}\omega /2{\nu }_{D}^{2}$ characterizes the different valley Hall insulators separated by the Dirac semimetal phase with *m* = 0 in the phase diagram. And Δ*ω* is the bandwidth for the opening of the full band gap, which is evolved with Δ*r* as shown in Fig. 2(a). It is indicated that the effective Hamiltonian is strongly dependent on the parameter Δ*r*. Utilizing the eigenvector from the effective Hamiltonian in equation (1), the local Berry curvature centered at K valley can be calculated^40^

2 $${{\rm{\Omega }}}_{K}(\delta {\boldsymbol{k}})=\frac{m{\nu }_{D}}{2{(\delta {k}^{2}+{m}^{2}{\nu }_{D}^{2})}^{3/2}}$$

Therefore, the topological charges of the first band can be calculated by integrating the local Berry curvature in half of the Brillouin zone^41, 42^

3 $${C}_{K}=\frac{1}{2\pi }\int {{\rm{\Omega }}}_{K}({\delta }{\boldsymbol{k}})dS=\frac{1}{2}\mathrm{sgn}(m)$$

The Chern number of K′ valley can be similarly derived from time-reversal symmetry. Thus, the K′ valley also carries the equal topological charges with opposite signs ${C}_{K\text{'}}=-\frac{1}{2}\mathrm{sgn}(m)$. Obviously, when the parameter Δ*r* satisfies the conditions of Δ*r* < 0 or Δ*r* > 0, each valley carries a nonzero topological charge with the opposite sign, which leads to the nonzero topological invariant: valley Chern number C_*v*_ = (C_K_ − C_K′_) ≠ 0. For Δ*r* < 0 and Δ*r* > 0, the topological charge distributions at the K and K′ valleys are totally different and are summarized in Fig. 2(b). For Δ*r* < 0, the topological charge at the K valley is +1/2 and the topological charge at the K′ valley is −1/2, which results in a positive valley Chern number C_*v*_ = (C_K_ - C_K′_) = 1. On the contrary, for Δ*r* > 0, the topological charge at the K valley is −1/2 and the topological charge at the K′ valley is +1/2, which makes a negative valley Chern number C_*v*_ = (C_K_ − C_K′_) = −1. Therefore, the topological phase transition accompanied with the change of topological charge and valley Chern number occurs when the Δ*r* is equal to zero, which gives potential to valley dependent edge states by interfacing two hexagonal lattices with opposite Δ*r* parameters. Furthermore, for the second and third pairs of Dirac point degeneracies, the above conclusions are still applicable.

### Simultaneous multi-band topological edge states

**Figure 3:**
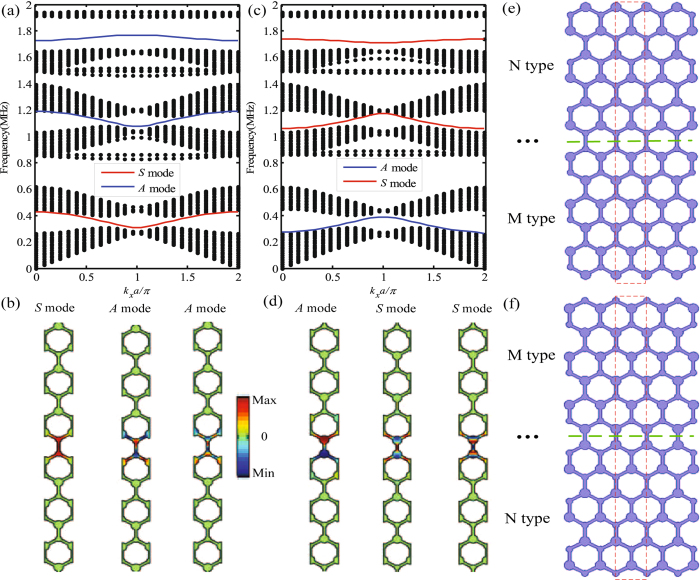
Simultaneous multi-band valley-protected topological edges states. (**a**) Calculated band structure of a super-cell consisting of two types lattices with Δ*r* = 0.06 at the top and Δ*r* = −0.06 at the bottom, namely NM type domain wall. (**b**) The stress field distributions of the three topological edges states and the schematic of their symmetries for NM type supercell. (**c**) Calculated band structure of super-cell consisting of two types lattices with Δ*r* = −0.06 at the top and Δ*r* = 0.06 at the bottom, namely MN type domain wall. (**d**) The stress field distributions of the three topological edges states and the schematic of their symmetries for MN type supercell. (**e**,**f**) Schematic of the corresponding structure of supercell for NM type and MN type, respectively.

To observe the valley-dependent edge state, we focus attention on the behavior at the interface between two hexagonal lattices with Δ*r* = 0.06 and Δ*r* = −0.06. As analyzed above, a hexagonal lattice with Δ*r* = 0.06 (Δ*r* = −0.06) has topological charge of −1/2 (+1/2) at the K valley and +1/2 (−1/2) at the K′ valley. The local topological charge differences across the edge at the K and K′ valleys are +1 and −1, respectively, which also corresponds to two different types of N and M visualized by the insets in Fig. 2(a). Thus, when the domain wall is constructed by different combinations of NM type and MN type, simultaneous multi-band topological edge states should emerge at the interfaces. Figure 3 shows the projected band structures and field distributions along the Г-K direction for the constructed supercell of NM type and MN type. The corresponding structures of the super cell are displayed in Fig. 3(e) and (f). For NM type supercell with Δ*r* = 0.06 at the top and Δ*r* = −0.06 at the bottom, there are three topological edge states appearing in the complete band gaps as shown in Fig. 3(a). It can be seen that three edge states occupy three frequency bands from 0.305 to 0.426 MHz, 1.073 to 1.195 MHz and 1.726 to 1.768 MHz, respectively. In addition, it can be observed that the first edge state is the symmetric (*S*) mode and the second and third edge states are antisymmetric (*A*) modes, which are confirmed by the stress field distributions on the interface corresponding to each edge state as shown in Fig. 3(b). Meanwhile, when the two hexagonal lattices are reset with Δ*r* = 0.06 at the bottom and Δ*r* = −0.06 at the top, namely MN type, which also exhibits three topological edge states plotted in Fig. 3(c). Compared to the NM type, these three edge states show opposite symmetry in that the first edge state is *A* mode and the second and third edge states are *S* mode, which can be indicated by the stress field distributions as shown in Fig. 3(d). Similarly, for MN type, the three edge states share three frequency bands from 0.275 to 0.388 MHz, 1.058 to 1.176 MHz and 1.707 to 1.736 MHz, respectively. Besides, it is worth noting that the first and second pairs of the edge states are gapless but the third pair of the edge states are gapped in our system. Therefore, the simultaneous multi-band topological edge states of SV waves are achieved in the simple system, which is greatly significant in designing multi-band and multi-functional sound devices.

### Simultaneous multi-band valley-protected backscattering suppression

**Figure 4:**
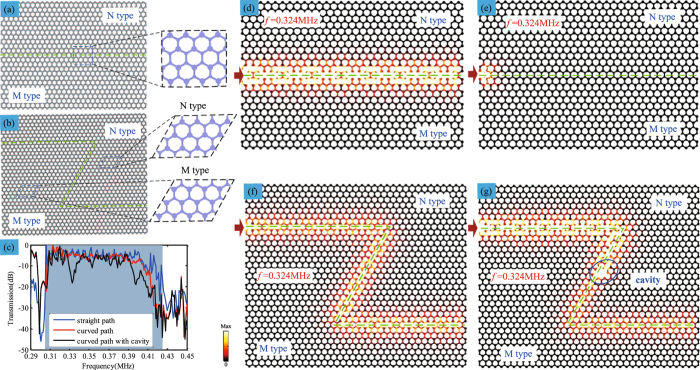
Valley-protected backscattering suppression of the topological edge states. (**a**,**b**) Schematic of the straight interface and Z-shaped interface, respectively, which are separated by two types of unit cells (M type and N type). (**c**) The transmission spectra of for the SV waves propagating through the straight path, curved path and curved path with cavities, respectively. (**d**,**e**) Stress field density distributions of the first *S* mode and *A* mode at the excited frequency of 0.324 MHz along the straight NM type interface, respectively. (**f**,**g**) Stress field density distributions of the first *S* mode at the excited frequency of 0.324 MHz along the curved NM type interface and that with a cavity, respectively.

It is noted that the simultaneous multi-band valley-protected topological edge states in our system stems inherently from the single-valley physics. One of the most important features of these topological edge states is the negligible backscattering in sharply curved phononic crystal interfaces. In the following, we further demonstrate the selective excitation of the edge modes and edge wave propagation around the constructed interfaces with a straight path, curved path and curved path with cavity, respectively. Due to the spatial parities of the valley-protected edge states, the excitation of edge modes exhibits a particular selectivity^24^. In our system, we find that only the *S* modes can be stimulated and that no *A* modes will not be excited. For the *A* modes, the two separated pseudospin states are mirror-symmetric in the horizontal direction but the *A* edge states are locally antisymmetric in each unit cell, which makes the *A* modes impossible to couple with the normally incident waves. Figure 4(a) and (b) display the schematic of the straight interface and Z-shaped interface, respectively, which are separated by two types of unit cells (M type and N type). When a point source is placed at the left side at an excited frequency of 0.324 MHz, the first *S* mode can propagate along the constructed edge of NM type while the first *A* mode is completely blocked as shown in Fig. 4(d) and (e), respectively. Furthermore, the valley-protected backscattering suppression around the Z-shaped curved waveguide is further demonstrated by the *S* edge modes. From Fig. 4(f), the first *S* mode is selected to be excited at the same frequency. It can be observed that the SV wave propagates along the sharply curved constructed edge, which also confirms the negligible backscattering of valley-protected edge states against a sharply curved interface. When a defect is introduced into the sharply curved edge as shown in Fig. 4(e), the SV wave can still go around the defect at the same frequency of 0.324 MHz. Besides, the transmission spectra of for the SV waves respectively propagating through the straight path, curved corners and curved corners with cavities are displayed in Fig. 4(c).

**Figure 5:**
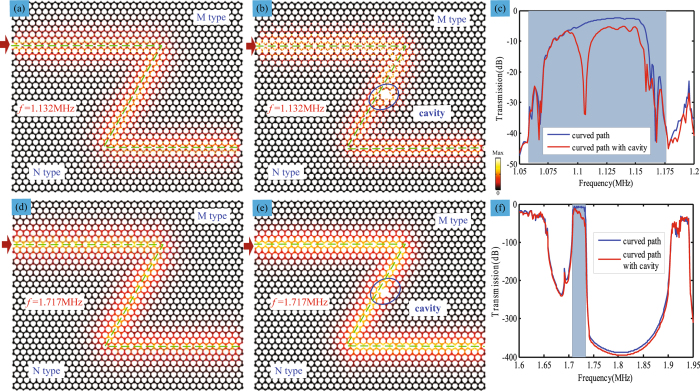
Simultaneous multi-band valley-protected backscattering suppression of the topological edge states. (**a**,**b**) Stress field density distributions of the second *S* mode at the excited frequency of 1.132 MHz along the curved MN type interface and that with a cavity, respectively. (**d**,**e**) Stress field density distributions of the third *S* mode at the excited frequency of 1.717 MHz along the curved MN type interface and that with a cavity, respectively. (**c**,**f**) The corresponding transmission spectra propagating along curved interface and that with cavity for the second and third *S* mode, respectively.

Finally, the valley-protected backscattering suppression of the multi-band topological edge states is further demonstrated. As shown in Fig. 5(a) and (b), when the second *S* mode is excited at 1.132 MHz, it also propagates along the curved edge and can go around the defect. For the third *S* mode, it exhibits the same behavior of negligible backscattering at the 1.717 MHz as shown in Fig. 5(d) and (e). Moreover, the corresponding transmission spectra of the second and third *S* mode are plotted in Fig. 5(c) and (f), respectively, which agree well with the band structures. So, the simultaneous multi-band valley-protected backscattering suppression of SV waves is successfully achieved. Moreover, the present topological acoustic system is efficient to encourage a practical study of experimental realization. Firstly, it is of great significance to manufacture the model, whose irregular shape made the actual processing a little difficult. On other hand, it is considered to enlarge the geometry size to reduce the frequency for the practical experiments and the suitable excitation device and amplifier to magnify the signal are essential, which will be the goal of our next research.

## Discussion

In summary, we have extended the concept of valley pseudospin into the SV waves in two-dimensional phononic crystals with veins. We have also demonstrated the simultaneous multi-band valley dependent edge states and the multi-band valley-protected backscattering suppression of SV waves. The topological properties originate from the topological transition induced by inversion asymmetry based on the parameter Δ*r*. The analysis of the local topological charge distributions and the opposite valley Chern numbers unveils the underlying topological transition. Such a platform achieves the multi-band valley dependent edge states of SV waves in two-dimensional structure, which is greatly significant to construct novel multi-band acoustic topological devices in sound signal detection, sound control and even other engineering fields.

## Methods

### Theoretical analysis

The physical design is carried out by constantly adjusting the geometric parameters (the radius of the steel rods *r* and the vein width *b*) to obtain three or more pairs of two-fold Dirac point degeneracies at K (or K′). The Dirac degeneracies are formed due to the protection C_*3v*_ symmetry. As the parameter Δ*r* (Δ*r* = (*r*_A_ − *r*_B_)/a) is varied, the inversion symmetry is broken and the Dirac degeneracies are removed, which produced a Dirac mass term of $m={\rm{\Delta }}\omega /2{\nu }_{D}^{2}$ in the effective Hamiltonian equation (1) based on the **k**•**p** perturbation method^24^. Δ*ω* is the bandwidth for the opening of the full band gap, which is strongly dependent with Δ*r* (Fig. 2(a)). As the parameter Δ*r* ranges from a negative value to a positive value, the topological charge distributions and the opposite valley Chern numbers are derived (Fig. 2(b)) by the equations (1), (2) and (3), which characterize the topological transition of the band diagram.

### Numerical calculations

All the numerical simulations are performed with COMSOL Multiphysics, a commercial package based on the finite element method. The acoustic properties of steel in the structures are set to 7780 kg/m^3^ for density and 3226.6 m/s for the speed of the transverse wave. Figures 1, 2 and 3 are calculated by using eigenfrequency studies. For bulk band structures of SV waves in Fig. 1(b), the periodic boundary conditions are imposed along the edges of the veins to form an infinite honeycomb lattice. For bulk projected band structures in Fig. 3(a) and (c), the supercell consists of 20 × 1 M type and N type unit cells and the periodic boundary conditions are imposed along the left and right edges of the supercell. Figures 4 and 5 are simulated by using frequency domain studies, in which the perfect matched layers are set surrounding the phononic crystal structures to avoid the influence of reflected waves. In addition, a sandwich structure by cladding M type and N type lattices might be helpful to make the edge states more tighten propagating along the domain wall, which could prevents possible edge states from leaking into free space.
